# Inoperable inflammatory myofibroblastic tumour of the para-nasal sinuses and orbit with recurrence responding to methotrexate and prednisolone: a case report

**DOI:** 10.1186/s13104-015-0993-3

**Published:** 2015-02-04

**Authors:** Mitrakrishnan Rayno Navinan, Isurujith Liyanage, Sandamalee Herath, Jevon Yudhishdran, Chrishan Shivanthan, Dulani Beneragama, Aruna Kulatunga

**Affiliations:** National Hospital of Sri Lanka, Colombo, Sri Lanka; Department of Pathology, Faculty of Medicine, University of Sri Jayawardenepura, Colombo, Sri Lanka

**Keywords:** Inflammatory myofibroblastic tumour, Prednisolone, Methotrexate, Para-nasal sinuses

## Abstract

**Background:**

Inflammatory myofibroblastic tumour is a rare neoplasm with a potential to behave in a malignant manner. It can occur anywhere in the body, however involvement of the head, especially the para-nasal sinuses is rare.

**Case presentation:**

A 33-year-old South Asian male presented with coryzal symptoms including a persistent cough with an asymmetrical swelling of the left side of the face. Imaging revealed a mass lesion involving the para-nasal sinuses eroding into the orbit. Histology and the clinical picture were compatible with inflammatory myofibroblastic tumour. As curative excision of the tumour was not feasible, medical management was offered. Despite early features of remission to glucocorticoids, tapering resulted in recurrence. Hence combination therapy with glucocorticoids and methotrexate was commenced with dramatic reduction of tumour burden and the patient has been in remission to date.

**Conclusion:**

Inflammatory myofibroblastic tumour has the potential to behave in a malignant manner. Medical management with chemotherapy, glucocorticoids and non-steroidal anti-inflammatory drugs though effective, do not have a uniform response pattern. Surgically unresectable inflammatory myofibroblastic tumour above neck should be treated aggressively with combination regimens. Combination of prednisolone with methotrexate has been shown to have good outcome.

## Background

Inflammatory myelofibroblastic tumour is a rare neoplasm with a variety of outcomes. Though considered histologically benign, it could demonstrate a course similar to a malignancy by way of invasion, recurrence and aggressive growth culminating in fatalities [1,2]. The commonest organ to be involved is the lungs. Other regions include the abdominal cavity, retroperitoneal region and bladder. However, virtually any organ can be involved. Above neck occurrence is uncommon, and involvement of the nasal and para-nasal cavities though rare, has been previously reported [1,3,4]. The clinical picture and imaging findings of inflammatory myelofibroblastic tumour could be confusing and often misinterpreted as a malignancy. Hence diagnosis and treatment could be challenging [5]. We report a case of a surgically unresectable Inflammatory myelofibroblastic tumour of the para-nasal sinuses with involvement of the orbit and temporal bones with recurrence after initial response to glucocorticoid mono-therapy, and subsequent sustained remission with combination of glucocorticoid and methotrexate.

## Case presentation

A 33 year old South-Asian male presented with coryzal lower respiratory tract symptoms, which was treated as atypical pneumonia in the context of initial unremarkable laboratory studies. Despite treatment the patient remained persistently symptomatic with a prominent and troublesome cough. Repeated laboratory investigations including chest X-rays were unremarkable. Symptoms persisted despite repeated courses of treatment as for sinusitis and allergic rhinitis complicated by a postnasal drip. He progressed to develop a unilateral facial swelling involving the left temporal, orbital and maxillary territories with trismus. Examination revealed facial asymmetry, with non-tender prominence of the left maxillary and ophthalmic territories of the face. General examination and systems examinations were otherwise normal with notable absence of lymphadenopathy or chest signs.

Laboratory studies revealed persistently elevated inflammatory markers with the lowest documented C - reactive protein level of 96 mg/dL and erythrocyte sedimentation rate of 132 mm in the first hour. Whole blood analysis demonstrated normal red cell indices (haemoglobin of 11.2 g/dL) with mildly elevated white cell count of 13.48 × 10^9^/L [4-11] which was predominantly neutrophilic - 67.8%. Blood film did not yield any additional information. Liver and renal biochemistry were normal. Antibody studies for C anti- neutrophil cytoplasmic antibody, P anti- neutrophil cytoplasmic antibody and anti-nuclear antibodies were negative. Quantiferon tuberculosis gold test was negative as were 3 serial samples of sputum for acid fast bacilli. Mycoplasma antibody titres were non-significantly elevated with a stable titre of 1/80.

**Figure 1:**
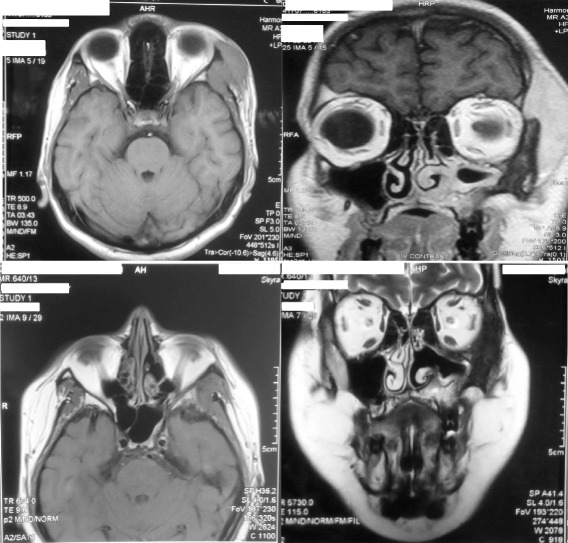
**Magnetic resonance images demonstrate the initial appearance and response to glucocorticoid mono-therapy.** Top row - Imaging of the head and face revealed abnormal thickening of temporalis. Asymmetry and loss of normal facial planes were seen with infiltrates noted in the left infra temporal fossa. Inflammatory changes are also seen in the ethmoid sinus , left maxillary sinus with muco-periosteal thickening and with non-uniform thickening and sclerosis of sinus wall with erosion postero-laterally. Extension of tumour was noted in the left orbit antero-laterally with lacrimal gland involvement. Bottom row- Imaging demonstrates the initial significant response to steroid monotherapy which prompted rapid tailing off.

Magnetic resonance imaging (MRI) of the head and face revealed abnormal thickening of left sided temporalis, pterygoid and masseter muscles. Asymmetry and loss of normal facial planes was seen with invasion into the left infra temporal fossa and left para-pharyngeal space with encirclement of the carotid artery. Inflammatory changes were seen in the left maxillary sinus with muco-periosteal thickening and with non-uniform thickening and sclerosis of sinus wall with erosion postero-laterally. Inflammatory changes were seen in the ethmoid sinus as well. The left orbit showed invasion antero-laterally with lacrimal gland involvement (Figure 1). The right side of the face was completely spared. Contrast enhanced computer tomography (CT) of the chest, abdomen and pelvis ruled out concomitant or metastatic lesions.

**Figure 2:**
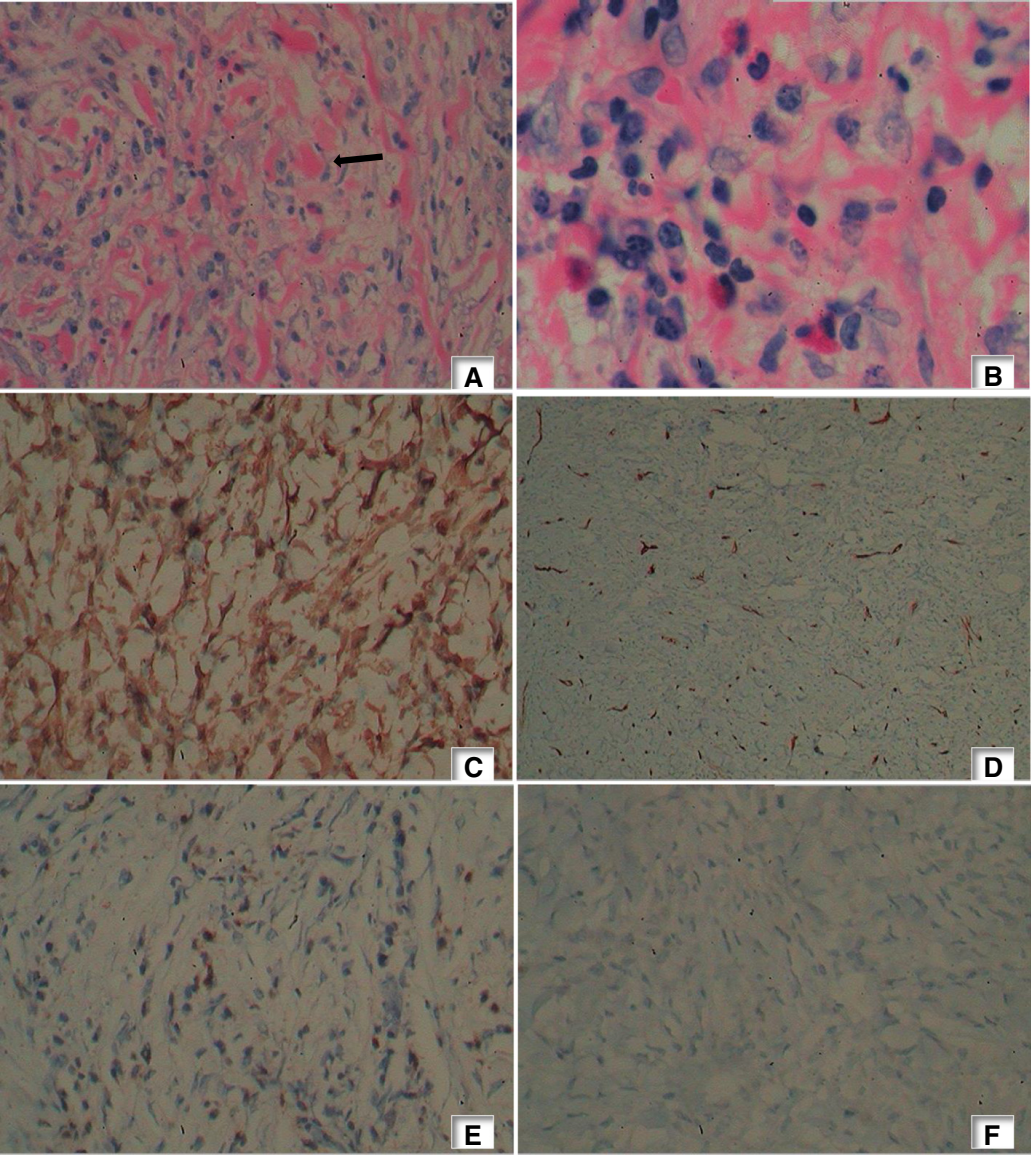
**Histological appearance and immunohistochemistry staining of the tumour. A**- Spindle cells and inflammatory cells mixed with collagen (arrow) (Haematoxylin and Eosin 40 x10), **B**- Spindle cells with a mixed population of inflammatory cells composed of lymphocytes, histiocytes, eosinophils and plasma cells in a collagenous background (Haematoxylin and Eosin 100 x10), **C**- Vimentin- Diffuse, strong positivity, **D**- Cluster of difference 34- Spindle cells negative , **E**- Leukocyte common antigen - Patchy positivity highlighting the lymphoid cells; other cells negative , **F**- Anaplastic lymphoma kinase 1- Negative.

Biopsy of the left orbital wall region mass demonstrated spindle cells, collagen and inflammatory cells. The spindle cells and collagen were arranged in an admixture of both a scar like pattern and a vague storiform pattern. There was a moderate to heavy diffuse polymorphic infiltrate consisting of plasma cells, histiocytes, eosinophils and lymphocytes. Mitotic activity was sparse. Significant atypia or necrosis were notably absent. Immuno-histochemistry was positive for both smooth muscle actin (SMA) and vimentin antibodies in the spindle cells. Leuckocyte common antigen (LCA) positivity was seen only in the surrounding lymphoid cells. Immunohistochemistry studies were negative for Melan A, Cluster of differentiation(CD)34, anaplastic lymphoma kinase 1 (ALK), CD 30, CD 20 and CD 1a( Figure 2). Biopsy of the left temporal region showed fibro fatty tissue with scattered infiltrates of lymphocytes with a population of plump spindle cells. No mitotic activity or cellular atypia were present. Histology and the immunohistochemistry results favoured a diagnosis of inflammatory myelofibroblastic tumour. Biopsy studies for tuberculosis and fungal filaments were negative.

**Figure 3:**
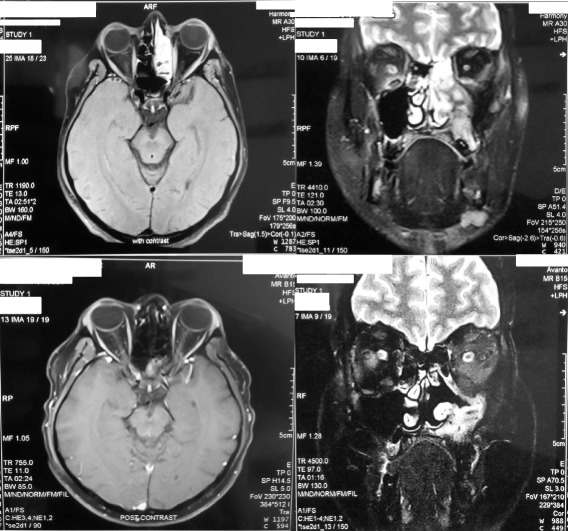
**Magnetic resonance images show response with combination therapy of methotrexate and prednisolone.** Top row - Imaging reveals the recurrence of tumour in the left temporal region and infra temporal fossa with involvement of the sinuses [left maxillary antrum projecting into the middle meatus of the nose, sphenoidal and ethmoid sinus] and involvement of the recti muscles of the left orbit. Bottom row- Imaging demonstrates absence of tumour in the temporal region and orbit and clearance within the middle meatus. Though reduced, persistence of residual tumour within the left maxillary antrum is seen.

The patient was initially commenced on a high dose of oral prednisolone which resulted in symptomatic improvement with follow-up MRI evidence of overall reduction of tumor mass (Figure 1). The paucity of literature guidance or formal guidelines for treatment resulted in premature taper and withdrawal of steroids over 6 months. Despite the initial remission, the patient developed features of recurrence characterized by worsening symptoms similar to that of the initial presentation symptoms with multiple episodes of left sided epistaxis. Fibre-optic endoscopic assessment of the nasal cavity revealed a pale, friable polypoidal mass with contact bleeding originating from the left maxillary sinus. MRI Imaging confirmed the recurrence of the tumour in the left temporal region and infra temporal fossa with involvement of the surrounding sinuses and the left maxillary antrum projecting into the middle meatus of the nose with involvement of medial, lateral and inferior recti in the left orbit with definite enhancement of the dura overlying the left middle cranial fossa (Figure 3). A prompt diagnosis of tumor recurrence was made and treatment was re commenced with both high dose prednisolone and once weekly low dose methotrexate with very slow taper of prednisolone after sustained remission and continuation of methotrexate. After dual agent treatment for 6 months an MRI was repeated which showed virtually total regression of tumour in the temporal region and orbit , and minimal residual tumour within the left maxillary antrum ( Figure 3). The patient is currently on the same treatment with the intent to slowly taper the prednisolone to a low maintenance dose along whilst continuing methotrexate until satisfactory and sustained disease control is achieved guided by radiological and clinical response on periodic reassessment.

## Discussion

The diagnosis of inflammatory myofibroblastic tumour, is often overlooked and missed because it is very rare. Symptoms are often non-specific though they could be related to the anatomical location of IMT and mass effect [6]. Recurrent bleeding manifestations by way of epistaxis, haematuria and rectal bleeding [1,6,7] have been observed and attributed to the vascular nature of the neoplasm [1]. However this manifestation alone has little diagnostic value. In the absence of definite histology a variety of differential diagnoses could have been entertained in the case subject including Wegner’s granulamatosis, lymphoma, opportunistic infections such as rhinocerebral mucormycosis and tuberculosis. Imaging was helpful to identify a neoplastic process as a result of prominent local infiltration. The value of histology in the diagnosis of this condition is quite apparent.

Follicular dendritic cell (FDC) tumour is an equally rare differential diagnosis that should be entertained as a result of stark similarity in histology. Nonetheless the clinical picture of FDC tumour is significant for lymphadenopathy with involvement of the head, neck, mediastinum and axilla in the context of a painless mass lesion most importantly in the absence of significant constitutional symptoms. However involvement of extra-nodal reticulo-endothelial sites have been reported including tonsils, spleen and gastrointestinal mucosa associated lymphoid tissue [4]. Immuno-histochemistry is useful to separate FDC from IMT, as CD 21,23 and 35 are positive only in FDC. Other useful bio markers include R4/23, Ki-FDC1p, KiM4 and clusterin which are seen in FDC [8]. These markers are currently not available in Sri Lanka and hence could not be performed in the case subject. Anaplastic lymphoma kinase reactivity, which was negative in our patient, is known to be seen in approximately 56% of cases of IMT, and when present has prognostic value by way of lower likelihood of metastases however a greater probability of recurrence [9] and favors a diagnosis of IMT over FDC. Spindle cells in IMT also show positivity for vimentin and smooth muscle actin, both of which were seen in the case subject. However vimentin may also stain positive in FDC. SMA positivity which was noted in the case subject is common in IMT though not common in FDC, its presence does not exclude it completely [4,10-13]. Though the absence of comprehensive immunohistochemistry panel studies was an impediment in establishing an irrefutable diagnosis of IMT, the absence of involvement of structures typical for FDC including nodal and extranodal sites both clinically and on computed tomography imaging and the presence of prominent constitutional symptoms and typical histology (presence of spindle cells associated with infiltrates of mononuclear inflammatory cells such as plasma cells, lymphocytes, histiocytes) and available immunohistochemistry findings favoured and supported the diagnosis of IMT and excluded FDC [4].

Medical literature shows confusing, overlapping and interchangeable use of the terminologies IMT and IPT (inflammatory pseudo tumour) and this is an area of debate as both terms describe pathologies with very similar clinical and histological presentations. IMT is defined by World Health Organization as a distinct borderline lesion composed of myofibroblastic cells with a variable admixture of inflammatory cells, and the terms IPT and IMT are considered synonymous [14]. However some authorities in the subject consider IPT as an overall broader category, and IMT as a subset of IPT that can show prominent neoplastic features [15], though considered histologically benign [16]. Despite numerous similarities in histology, subtle differences such as marked spindle cell proliferation in IMT and prominent lymphoplasmacytic infiltrates in IPT help to histologically differentiate them. Immunohistochemical markers such as IgG4-positive plasma cells and an increased ratio of IgG4+/IgG+ are invaluable in differentiating IPT from IMT, where these are not significantly present. ALK exclusivity to IMT is invaluable in differentiation from IPT [17]. In the index patient, cellular atypia, atypical mitosis and pleomorphism favoring malignant transformation were not seen [9]. However, radiological evidence of sino-nasal destruction exemplifies the malignant tendencies of the lesion despite benign histology [3]. Similarly tumour recurrence also is a feature of malignant neoplastic tendencies. The mixed benign and malignant features of the neoplasm and the observed remission to initial chemotherapy and relapse of on withdrawal is keeping in line with the poorly understood characteristics of the lesion [7]. Furthermore, IMT originating from extra-pulmonary regions is known to show a more aggressive behaviour [4].

Management of IMT can be challenging as there are no established treatment protocols [1]. Surgery is considered first line for resectable lesions and may be combined with alternate regimens such as corticosteroids, radiotherapy or chemotherapy [3]. Radical surgical excision has shown to be curative in 90% of the cases [18]. However the anatomical location and proximity to vital structures may preclude surgical excision [19]. Mortality is well documented despite optimal multimodal therapy [2,20]. Glucocorticoids are considered more effective in children than in adults [16] however literature evidence in adults shows good response to high dose glucocorticoid mono-therapy with rapid induction of remission [21,22]. Multi agent combination therapy regimens have been suggested for unresectable tumors’ [23], however their value in the treatment of IMT is controversial [24]. There is little literature guidance on treatment duration with glucocorticoids and success has been reported with treatment durations spanning from anywhere between 6 months to years [4,8,3]. Variable response to long term use of steroids as well as complete remission following weaning of steroids have been noted [8]. Steroid dependence to maintain remission is a therapeutic problem often encountered in the treatment of IMT [12]. Steroid refractory IMT requiring alternate agents is well documented [13]. Paradoxically emergence of IMT whilst on immunosuppression following kidney transplant has been reported putting into perspective the basis of immunosuppressive treatment in the management of IMT [11]. The definitive role of glucocorticoid therapy in IMT is not clearly understood but a therapeutic trial should be considered in appropriate cases and continued into remission if clinical and radiological response is apparent.

Chemotherapy is recommended when IMT is multifocal, invasive or shows local recurrence [16]. It is often a combination of agents including methotrexate, cisplatin, vinorelbine, Adriamycin®, carboplatin and paclitaxel given with a view of achieving complete remission [13,15,17,23,25]. These agents have also been used as an adjunct with non-steroidal anti-inflammatory drugs and glucocorticoids and combination therapy has shown good response in instances where mono-therapy had failed [13]. A change in the combination of chemotherapeutic agents has been shown to be beneficial when the initial choice of combination agents have failed to yield good response [17]. Chemotherapy can also be utilized as neo-adjuvant treatment prior to definitive surgical intervention [14]. Non-steroidal anti-inflammatory drugs used alone has also demonstrated success [26]. Immunomodulation with intravenous immunoglobulin therapy has been tried with success for post-surgical residual tumour with successful long term remission [27]. Another novel therapy under evaluation is the ALK inhibitor crizotinib, which showed long term partial response in those who were ALK positive [28]. Radiotherapy is usually considered ineffective at standard doses. Response with high doses is purported, however the side effects and complications may be a limiting factor [29].

The variable response to glucocorticoids and other forms of chemotherapy, with non-uniformity in response to similar regimens and the use of agents as individualized therapeutic trials have their foundation in the lack of clinical evidence to guide management. The anatomical sites of involvement and the involvement of critical structures and the side effects of classical chemo-radiotherapy fuelled by the favourable response to glucocorticoid mono-therapy, guided us to opt for combination chemotherapy with steroids and methotrexate. Clinical, radiological (Figure 3) and inflammatory marker response were helpful in adjusting dosage of treatment.

The prognosis in IMT is generally good [10]. Precise prognostic predictors are yet to be established. However surgical resectability with complete resection, tumour size and ALK expression [16,20,30] are variables that have been known to alter prognosis and disease evolution. Recurrence is known to be associated to site, size, age and incomplete resection [31,32]. Tumour recurrence after surgery was found to be as much as 25-37% in extra-pulmonary IMT [3,18] and up to 85% in abdomino-pelvic IMT [9]. IMT recurrence in nasal sinuses is common with usually an aggressive course, and may be influenced by histology [3] though it is a subject of much debate [31]. Long term follow up is important as the condition has been reported to recur many years after apparent cure [10].

## Conclusion

Inflammatory myofibroblastic tumor is a poorly understood benign pathological disease process which has the propensity to mimic a malignancy due to local invasion and growth. Immunohistochemistry and histology are essential for diagnosis and commencement of early and appropriate treatment. Curative surgery is preferred if the anatomical location and surrounding structures involved are permissible. Though trial evidence for medical treatment is lacking for routine recommendation in inoperable patients anecdotal evidence and our experience suggest that it is a reasonable alternative with possible favourable outcome. Medical treatment should be guided by clinical and radiological response. Our experience demonstrates that glucocorticoids with methotrexate in combination maybe an effective regimen for surgically unresectable inflammatory myofibroblastic tumour. Duration and intensity of treatment duration should be individualized guided by clinical and radiological response.

## Consent

Written informed consent was obtained from the patient for publication of this Case Report and any accompanying images. A copy of the written consent is available for review by the Editor-in-Chief of this journal.

## Abbreviations

MRI: Magnetic resonance imaging
CD: Cluster of difference
CT: Computed tomography
LCA: Leukocyte common antigen
IMT: Inflammatory myofibroblastic tumour
IPT: Inflammatory pseudo tumour
ALK: Anaplastic lymphoma kinase 1
FDC: Follicular dendritic cell tumour
SMA: Smooth muscle actin
